# Cat predation of Kangaroo Island dunnarts in aftermath of bushfire

**DOI:** 10.1038/s41598-022-11383-6

**Published:** 2022-06-16

**Authors:** Patrick Hodgens, Heidi Groffen, Ryan O’Handley, Ajai Vyas, Louis Lignereux

**Affiliations:** 1 Terrain Ecology, Kangaroo Island, Australia; 2 School of Animal and Veterinary Science, University of Adelaide, Adelaide, Australia; 3 School of Biological Sciences, Nanyang Technological University, Singapore, Singapore

**Keywords:** Biodiversity, Conservation biology, Invasive species, Biodiversity, Conservation biology, Invasive species

## Abstract

The Kangaroo Island dunnart (*Sminthopsis aitkeni*) is a critically endangered marsupial species with an estimated population of ~ 500 individuals found only on the western end of Australia's third largest island. Severe bushfires recently burnt more than 98% of its known and predicted habitat that was already under pressure from fragmentation. After the fires, we found evidence of eight individual dunnarts in the digestive tract of seven feral cats, out of the 86 collected in remaining unburnt refugia; thus demonstrating the need of immediate risk management efforts after large-scale stochastic events.

## Introduction

Kangaroo Island (~ 4400 km^2^, KI hereafter) is the third largest island in Australia. It underwent substantial land clearing, and consequent fragmentation of the natural bushland habitat, after World War II^1,2^. Relatively intact western KI was eventually identified as a key biodiversity hotspot^3^, home to several endangered and endemic native species including the KI dunnart.

Dunnarts (*Sminthopsis* spp.) are small insectivorous dasyurid marsupials. The KI dunnart is distinguished from the other 17 dunnart species found in Australia by morphological features, including manus, pes, and penis shape^4^. This endangered species is the only dasyurid found on the island, exclusively resident in ~ 342 km^2^ before 2020^5^, and found nowhere else in the world^2^. The species is rarely recorded, with only 28 individuals found during > 33,000 trap-nights pre-2019^5^. With a low number of individuals restricted to a small geographic area, the KI dunnart is exceptionally vulnerable to stochastic events. Predation by feral cats (*Felis catus*) is likely to be another source of pressure on the KI dunnart. Cats were introduced to KI during European settlement and quickly became apex predators, reaching higher relative abundance than adjacent mainland^6^ with an estimated density of 0.37 ± 0.15 cat/km^2^^5^. Cat predation has been the cause for extinction or near-extinction of several native species around the globe^7^, with the extinction risk becoming increasingly acute in insular islands like KI. Cat predation on islands has contributed to > 13% of globally recorded extinction events, accounting for > 8% of instances within these taxa of species being pushed to critically endangered status^8^. A recent meta-analysis found evidence of cat predation for three critically endangered species and four endangered species in Australia on the IUCN Red List of Threatened Species^7^.

Australian bushfires in 2019–2020 burnt ~ 97,000 km^2^ of vegetation^9,10^, with damage overlapping with habitats of > 100 threatened species. Dry lightning storms in the remote and vegetated northwest of the Island started the bushfire in the KI. The bushfire eventually spread easterly, burning approximately 98% of the known and predicted habitat of the KI dunnart^10^.

In this study, we have analysed the diet of feral cats humanely euthanized in designated areas of local conservation interest immediately after the 2019 KI bushfire.

## Materials and methods

### Study site

**Figure 1:**
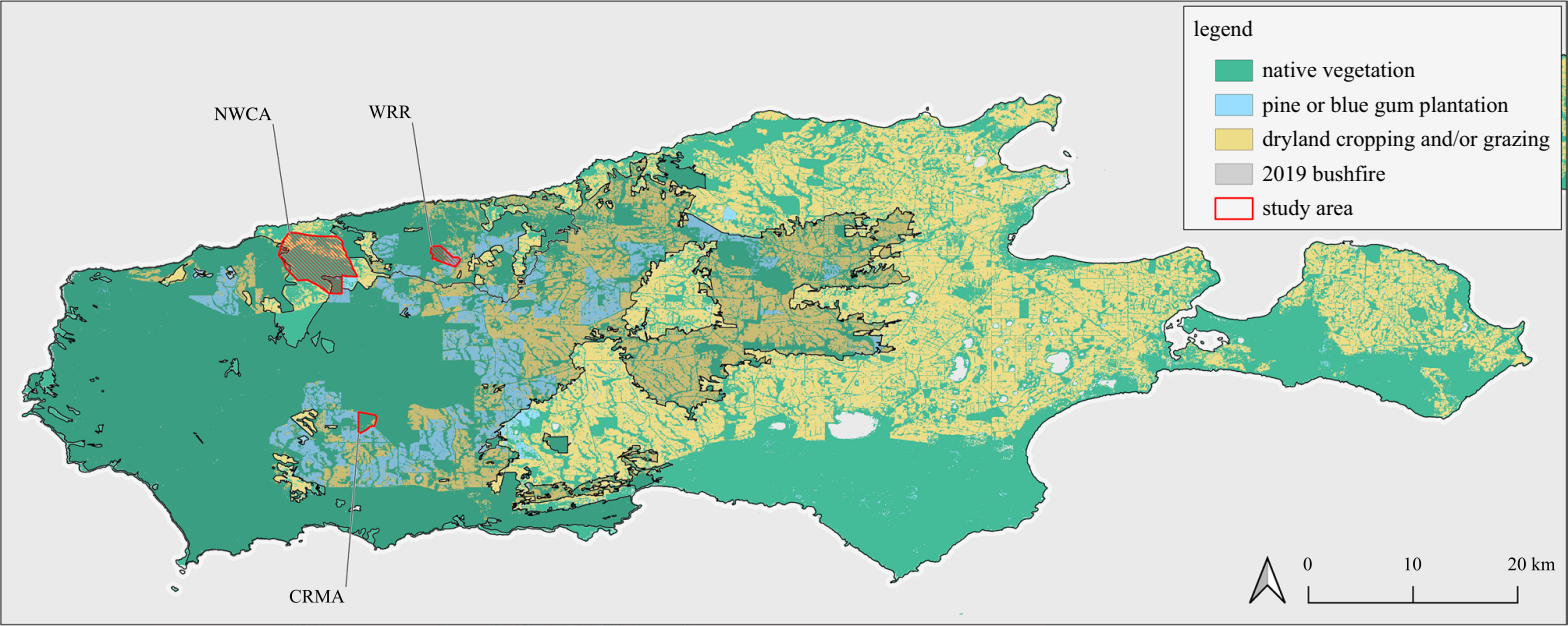
Map of Kangaroo Island. The three study areas (*CRMA* Church Road management area, *NWCA* North-West Conservation Alliance, *WRR* Western River Refuge) are shown in relation to native vegetation and unburnt refugia following the 2019 bushfire. The study areas with predated KI dunnart are hatched. We used QGIS 3.20 to elaborate the map (QGIS Development Team, 2021 QGIS Geographic Information System. Open-Source Geospatial Foundation Project, http://qgis.osgeo.org), with bushfire and vegetation datasets recovered from data.sa.gov.au.

Due to potential threats, non-government organization Kangaroo Island Land for Wildlife, and local landowners recommenced surveys for KI dunnart. Two priority areas were the small unburnt patch (0.12 km^2^) at the Western River Refuge and the much larger and completely unburnt 4.2 km^2^ patch of vegetation at the North-West Conservation Alliance within the De Mole River Catchment (locations depicted in Fig. 1). Feral cat control was commenced immediately at these critical sites, primarily through baited cage traps, live shooting, and the use of Felixer Grooming traps. Additional feral cat control was commenced at the Church Road Management Zone. This burnt region was a biological site of significance pre-fires containing many threatened species, including the KI dunnart.

### Cats used in this study

The study was based on cat tissue available from earlier conservation efforts. We accessed the stomach contents and digestive tracts of 86 cats that were captured between February–August 2020. At each of the management zones depicted in Fig. 1, cage traps were baited with chicken wings in afternoons and checked the following mornings. All cats were humanely euthanised by firearm. At the Western River Refuge a grid of between 40 and 60 traps were operational during this time. Another 40 to 50 traps at the North West Conservation Alliance and 25 traps were established at the Church Road Management Zone. The traps were set on an ad hoc basis soon after the fires and more strategically as the program progressed.

### Diet analysis

We collected stomach and large intestine of cats; placing the tissue in individual resealable plastic bags each identified with the cats pelage, sex, date and trap location. We dispatched frozen samples (− 20 °C) to a specialist lab for prey identification (ScatsAbout, Majors Creek, NSW). We found readily recognizable prey in gastric content, and confirmed their identification by comparison with reference bone collection. Digested mammalian prey in large intestine samples were washed and sieved and subsequently identified with hair identification techniques based on the overall profile and length of the hair, their medulla, cross-section, and cuticle scales casts patterns as described earlier^12^ and developed in the Hair ID software (Ecobyte, Australia). We made identification to the highest possible taxonomic status. We used Wald method to quantify 95% confidence intervals.

### Ethics declaration

Our study utilized tissues from feral cats that were killed for other purposes than obtaining these materials for scientific research. The feral cat is categorised a pest in Australia and undergoes a national control program (https://www.awe.gov.au/biosecurity-trade/invasive-species/feral-animals-australia/feral-cats). The feral cats were trapped and humanely euthanised in accordance with the South Australia Animal Welfare Act 1985 in an already ongoing conservation program. Institutional animal care and use committee approval was not required for scavenging the tissue of these feral cats, as per the prevailing guidelines from University of Adelaide.

## Results

We obtained stomach contents and digestive tracts of 86 cats trapped in February-August 2020.

**Figure 2:**
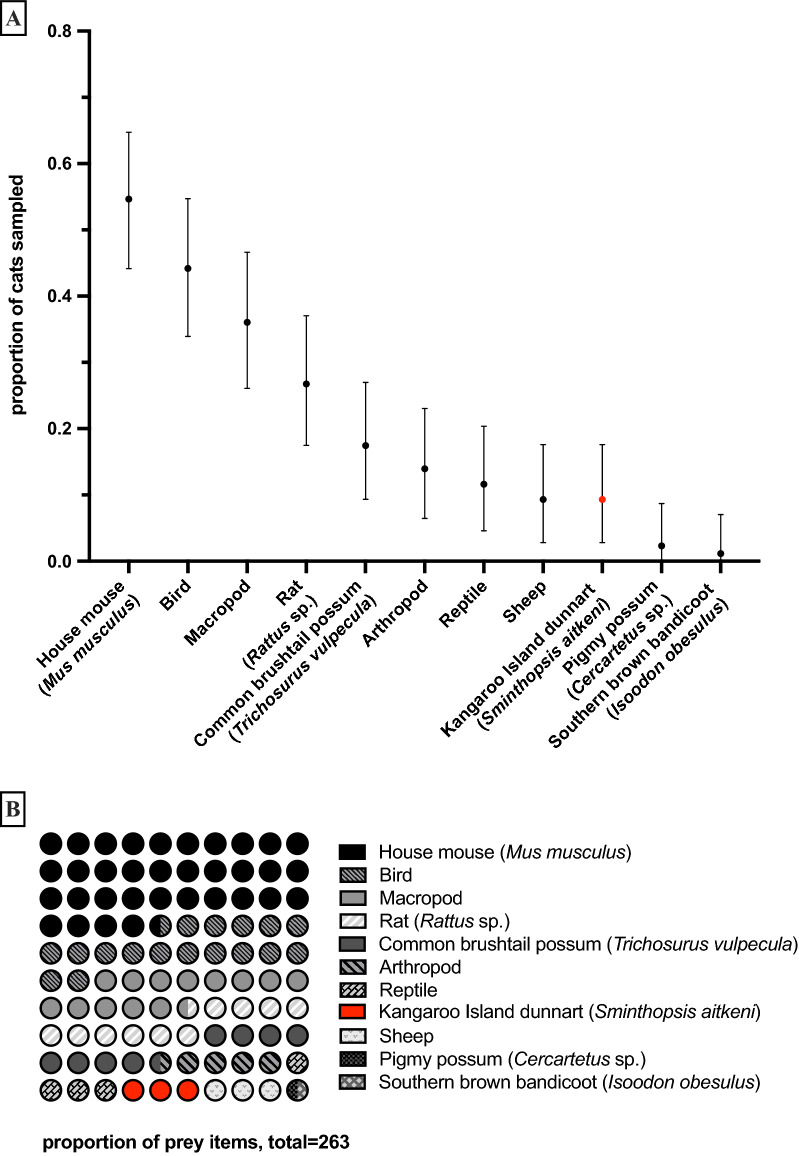
Cat predation of KI dunnarts. The top panel depicts broad categories of prey harvested from cat digestive tracts (abscissa) as a proportion of cats investigated (ordinate). The bottom panel depicts abundance of KI dunnart vis-à-vis total instances of preys recovered. Proportional contribution of KI dunnarts in the aggregate prey instances is depicted in the red.

We identified 263 distinct prey items (Fig. 2, Supplementary Table 1), including tetrapods (195 mammals, 46 birds, 10 reptiles) and 12 arthropods. Among them, the introduced house mouse (*Mus musculus*) represented the most significant proportion (36.3% of tetrapods; 95% CI 26.5–46.4), being part of diet for 47 cats (54.7%; 95% CI 44.2–64.7).

**Figure 3:**
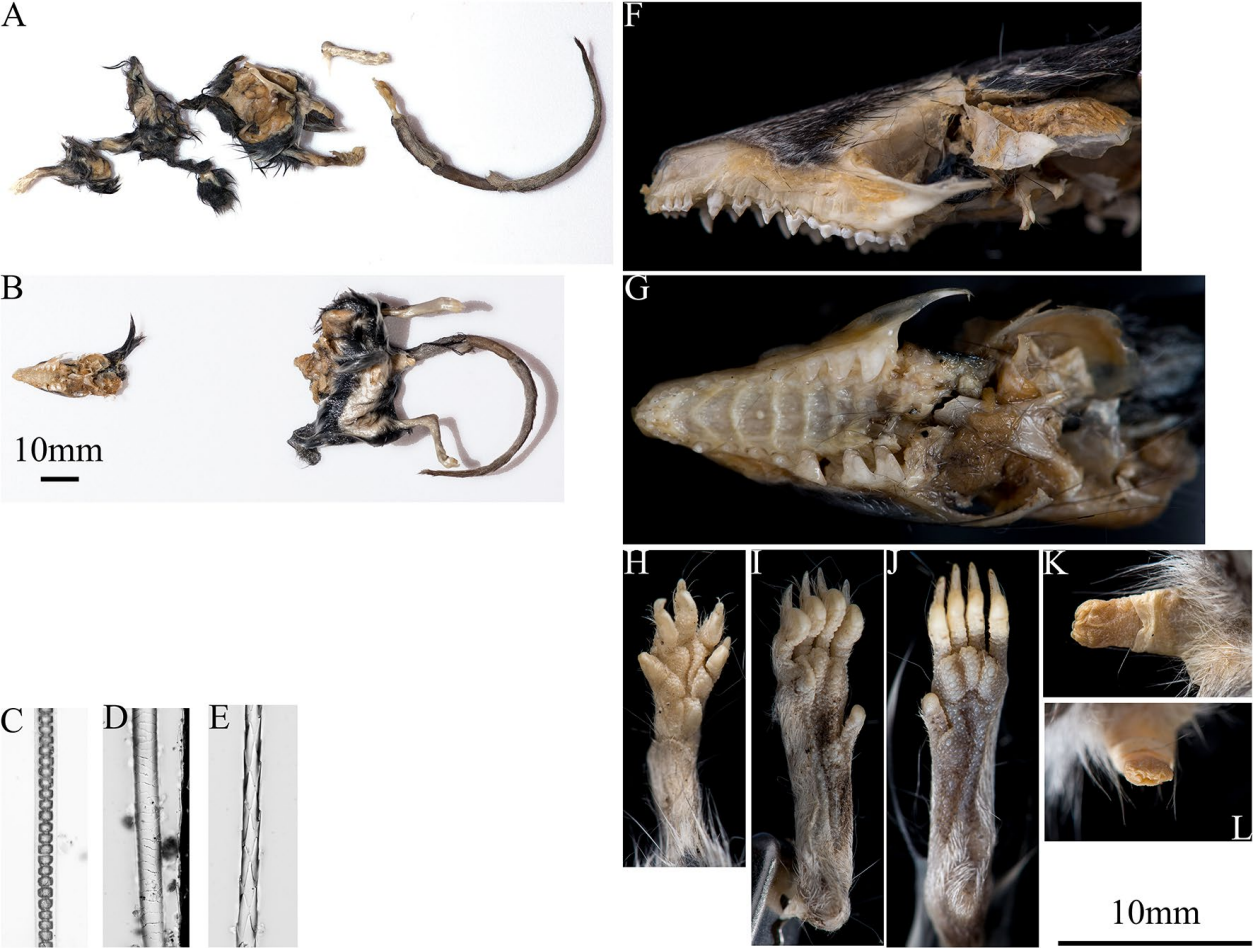
KI dunnart identification. Remains of two dunnarts removed from cat digestive tract are depicted in panel (**A**) and (**B**), with hair mounts depicted in panels (**C**) through (**E**). Lateral and maxillary occlusal view of the head can be seen in panels (**F**) and (**G**), respectively. Panels (**I**) and (**J**) depict manus and pes. Panels (**K**) and (**L**) depict dorsal and cranial view of penis, respectively. Photo credits: Louis Lignereux.

We observed the remains of eight KI dunnarts in seven different cats (Supplementary Table 2). Three dunnarts were readily identifiable through distinct anatomical features as they were nearly whole carcasses (Fig. 3). Five more instances of KI dunnart predation were identified based on characteristic hair features. We observed dunnart tissue in both stomach and large intestine of one cat, suggesting recent predation of at least two individuals.

Therefore, 8.1% (95% CI 3.8–16.2) of the sampled cats had predated on dunnart. These KI dunnarts were predated within the North-West Conservation Alliance at the DeMole River and the Western River Refuge (~ 10% of cats predating on dunnarts). No evidence of the dunnart was found in the remaining 19 cats trapped in the Church Road area. The KI dunnart comprised 3.2% (95% CI 0–7.9) of the recovered tetrapods prey items across three study areas.

We also found the remains of the endangered southern brown bandicoot (*Isoodon obesulus*) in a male cat's stomach (0.4% of tetrapods, 95% CI 0–3.4) at the Western River Refuge.

Birds were not identified down to the species level, and this group represented 18.3% of all tetrapod prey (95% CI 6.6–26.9), followed by macropods (13.9%; 95% CI 6.6–20.8).

## Discussion

Our results confirm for the first time that feral cats do predate on KI dunnart and that they were efficient hunters of this species directly after the fire. The high rates of predation also show that sampling of cat stomachs was a highly successful method of detecting the species. In comparison, scientific surveys in the earlier years resulted in much lower live capture rates (< 0.1% per trap-night, compared to 8.1% of cats)^5,9^, suggesting the relative ease by which cats were preying on this species, that had remained elusive to human surveying efforts. Diet analysis represents predation as a small snapshot of what the feral cat has recently consumed, restricted to the time before the prey is fully digested, estimated between 26.5 and 35.7 h^13^. Thus, cats present substantial predation pressure, especially for an endangered species with a low population size living in a small and degraded habitat.

We also found remains of southern brown bandicoot. This endangered species is likely the last out of eight native bandicoot species still extant in the wild in South Australia. The introduced red fox (*Vulpes vulpes*) is a known predator of the southern brown bandicoot^14^, therefore KI is an important refuge for this species because of the island's lack of fox predation.

These factors increase the urgency and continuity of feral cat control efforts in the immediate aftermath of bushfire especially in unburnt refugia on KI. Feral cats have been removed from more than four dozen islands worldwide^15^. Most of these islands have been small, with > 95% being less than 10 km^2^ in area. In comparison, 4400 km^2^ size of the KI presents a unique challenge to cat eradication efforts. In parallel to continuing the challenging efforts to remove feral cats from the large island^16^, it is critically important to sustain feral cat control at priority threatened species refugial sites.

## Conclusion

KI and its endemic dunnart species are emblematic of challenges faced by threatened species across the globe that are confined in increasingly fragmented habitats, coping with climate change's catastrophic consequences, and affected by introduced predators. These challenges act successively and cumulatively. Species already compromised by low population and small geographic range can easily slide into extinction during environmental catastrophes like the recent bushfires. While these events occur stochastically, their frequency and vigor have been augmented by anthropogenic influences resulting in increasingly drier and warmer weather conditions^17,18^. Mortality brought about by episodes like wildfire is especially damaging to endangered species with low population sizes, reducing effective population size and causing genetic bottlenecks. Therefore, efforts to provide immediate relief from an invasive predator, including the provision of refuge, is critical and can mean the difference between survival and extinction.

## Supplementary Information

Supplementary Legends.

Supplementary Table 1.

Supplementary Table 2.

## Data Availability

All data generated or analysed during this study are included in this published article (and its Supplementary Information files).
