# Supplementary material for: Cat predation of Kangaroo Island dunnarts in aftermath of bushfire
Source: Sci Rep. 2022 Jun 16;12:7272. doi: 10.1038/s41598-022-11383-6 (PMC9203781; doi:10.1038/s41598-022-11383-6)
Supplement: Supplementary file 1 — Supplementary Legends. [file 41598_2022_11383_MOESM1_ESM.docx]

Supplementary Table 1. A detailed view of prey items recovered from feral cat digestive tracts. The numbers denote the minimum number of prey from each relevant category.

Supplementary Table 2. Capture location and biological characteristics of cats found with Kangaroo Island dunnart in the digestive tract.
