# Supplementary material for: Cat predation of Kangaroo Island dunnarts in aftermath of bushfire
Source: Sci Rep. 2022 Jun 16;12:7272. doi: 10.1038/s41598-022-11383-6 (PMC9203781; doi:10.1038/s41598-022-11383-6)
Supplement: Supplementary file 3 — Supplementary Table 2. [file 41598_2022_11383_MOESM3_ESM.docx]

Suppl. Table: Capture location and biological characteristics of cats found with Kangaroo Island dunnart in the digestive tract, with the number of KI dunnart predated by each cat.

| **Date** | **Number of dunnarts** | **Latitude** | **Longitude** | **Location** | **Sex** | **Weight (kg)** |
| --- | --- | --- | --- | --- | --- | --- |
| 12/04/2020 | 1 | -35.747071 | 136.873191 | NWCA | Female | 2.9 |
| 2/05/2020 | 1 | -35.747071 | 136.873191 | NWCA | Male | 3.6 |
| 15/06/2020 | 1 | -35.722308 | 136.977126 | WRR | Male | 4.1 |
| 02/07/2020 | 2 | -35.747071 | 136.873191 | NWCA | Male | 3.5 |
| 08/07/2020 | 1 | -35.722308 | 136.977126 | WRR | Male | 4.7 |
| 07/08/2020 | 1 | -35.747071 | 136.873191 | NWCA | Female | 2.5 |
| 20/08/2020 | 1 | -35.722308 | 136.977126 | WRR | Female | 2.2 |
